# A strategy to eradicate well-developed Krebs-2 ascites in mice

**DOI:** 10.18632/oncotarget.7311

**Published:** 2016-02-10

**Authors:** Ekaterina A. Potter, Evgenia V. Dolgova, Anastasia S. Proskurina, Alexandra M. Minkevich, Yaroslav R. Efremov, Oleg S. Taranov, Vladimir V. Omigov, Valeriy P. Nikolin, Nelly A. Popova, Sergey I. Bayborodin, Alexander A. Ostanin, Elena R. Chernykh, Nikolay A. Kolchanov, Mikhail A. Shurdov, Sergey S. Bogachev

**Affiliations:** ^1^ Institute of Cytology and Genetics, Siberian Branch of the Russian Academy of Sciences, Novosibirsk 630090, Russia; ^2^ Department of Natural Sciences, Novosibirsk State University, Novosibirsk 630090, Russia; ^3^ The State Research Center of Virology and Biotechnology VECTOR, Novosibirsk 630559, Russia; ^4^ Institute of Clinical Immunology, Siberian Branch of the Russian Academy of Medical Sciences, Novosibirsk 630099, Russia; ^5^ LLC Panagen, Gorno-Altaisk 649000, Russia

**Keywords:** ascites Krebs-2, cyclophosphamide, extracellular dsDNA, tumor-initiating stem cells, repair

## Abstract

We describe the strategy, which allows curing experimental mice engrafted with Krebs-2 ascites. The strategy is based on the facts that i) Krebs-2 tumor-initiating stem cells (TISCs) are naturally capable of internalizing fragments of extracellular double-stranded DNA (dsDNA); ii) upon delivery into TISCs, these dsDNA fragments interfere with the on-going DNA repair process so that TISCs either die or lose their tumorigenic potential. The following 3-step regimen of therapeutic procedures leading to eradication of Krebs-2 ascites is considered. Firstly, three timed injections of cyclophosphamide (CP) exactly matching the interstrand cross-link (ICL) repair phases that lead to synchronization of ascites cells in late S/G2/M. Secondly, additional treatment of ascites 18 hours post each CP injection (at NER/HR transition timepoint) with a composite dsDNA-based preparation interfering with the NER and HR repair pathways, so that tumorigenic properties of ascites cells are compromised. Thirdly, final treatment of mice with a combination of CP and dsDNA injections as ascites cells undergo apoptotic destruction, and the surviving TAMRA+ TISCs arrested in late S/G2/M phases massively enter into G1/S, when they regain sensitivity to CP+dsDNA treatment. Thus, this regimen assures that no viable cells, particularly Krebs-2 TISCs, remain.

## INTRODUCTION

We report on the studies performed in the laboratory of induced cellular processes at the Institute of Cytology and Genetics, SB RAS (Novosibirsk, Russia) which aimed at developing an efficient protocol to eradicate Krebs-2 ascites in experimental mice. The protocol is based on two major sets of observations. In the recent papers [1, 2], we discovered and thoroughly characterized a previously unappreciated biological phenomenon in which poorly differentiated cells including TISCs are able to internalize fragments of extracellular dsDNA, and they do so natively, without any special transfection procedures. One can therefore use TAMRA-labeled dsDNA probe as a universal marker of stem cells of various origin (including TISCs). We also showed that dsDNA fragments that become internalized by the cells during the repair of interstrand CP-induced cross-links potently interfere with the repair process, thereby completely abrogating the tumor-inducing potential of the tumor graft [2].

These phenomena were highly suggestive of an opportunity to completely eradicate TISCs and so to reduce the graft tumorigenicity to zero. As a result, the tumor would be destroyed by the appropriate immune surveillance systems. The most intriguing point of this approach is that cancer cells would not be targeted via classic pathway-blocking mechanisms (which are easily overcome via activation of alternative pathways), rather they would be killed via targeting the integrity of chromatin. The linear order of chromatin would not be recoverable, as internalized dsDNA fragments would completely block the NER and HR stages of repair.

## RESULTS

### Historical review of basic experimental observations that underlied the development of the therapy targeting Krebs-2 ascites tumors

Our initial proof-of-concept study uncovered several basic effects of how dsDNA fragments internalized by TISC-like Krebs-2 cells interfere with the repair of interstrand DNA cross-links (ICLs) induced by CP pre-treatment. Specifically, the following dynamic parameters were quantified: i) time to development of ascites or solid grafts after the treatments; ii) percentage of committed cancer cells and TAMRA+ TISCs in Krebs-2 ascites after the treatments; iii) tumor regression, remission and relapse following *in situ* treatments of ascites. Our experimental platform allowed us to describe the temporal dynamics of ICL repair in Krebs-2 cells. It constitutes a cycle of three periods 12 hours each: accumulation of dsDNA breaks, latent period, repair period [2]. Next, we characterized the drugs and drug combinations that display therapeutic activity towards ascites tumors. These include CP, dsDNA and CP+dsDNA. DsDNA may be comprised of native human dsDNA, native salmon sperm dsDNA, cross-linked salmon sperm dsDNA, and a mixture of the three dsDNA types. During the ICL repair, we pinpointed the periods and timepoints that had the largest impact on targeting the ascites tumors by CP, dsDNA and CP+dsDNA combinations.

The outline of ascites grafting experiments and therapeutic regimens tested is provided below. Six-to-nine day old ascites (5-9 ml ascites fluid with 0.6-2.0×10^7 Krebs-2 cells) were used for engraftment experiments, whereas four-to-five day old ascites (1-1.3 ml of ascites fluid with 0.2-0.3×10^6 Krebs-2 cells in it) were used for *in situ* treatment experiments. CP was given i.p. to mice as 300 mg/kg body weight. Fragmented native DNA preparations (hDNA and ssDNA) and nitrogen mustard cross-linked DNA (ICL-hDNA and ICL-ssDNA) were administered i.p. hourly or bihourly at a dose of 0.5-1 mg/injection (a total of 6 mg DNA per mouse), or as a single dose of 6 mg/mouse or as a composite mixture. Further details on the timepoints when the preparations were given to mice are provided in the text or are schematically shown in the figures.

The following therapeutic effects have thus far been characterized.

### CP as a monotherapy

**Figure 1:**
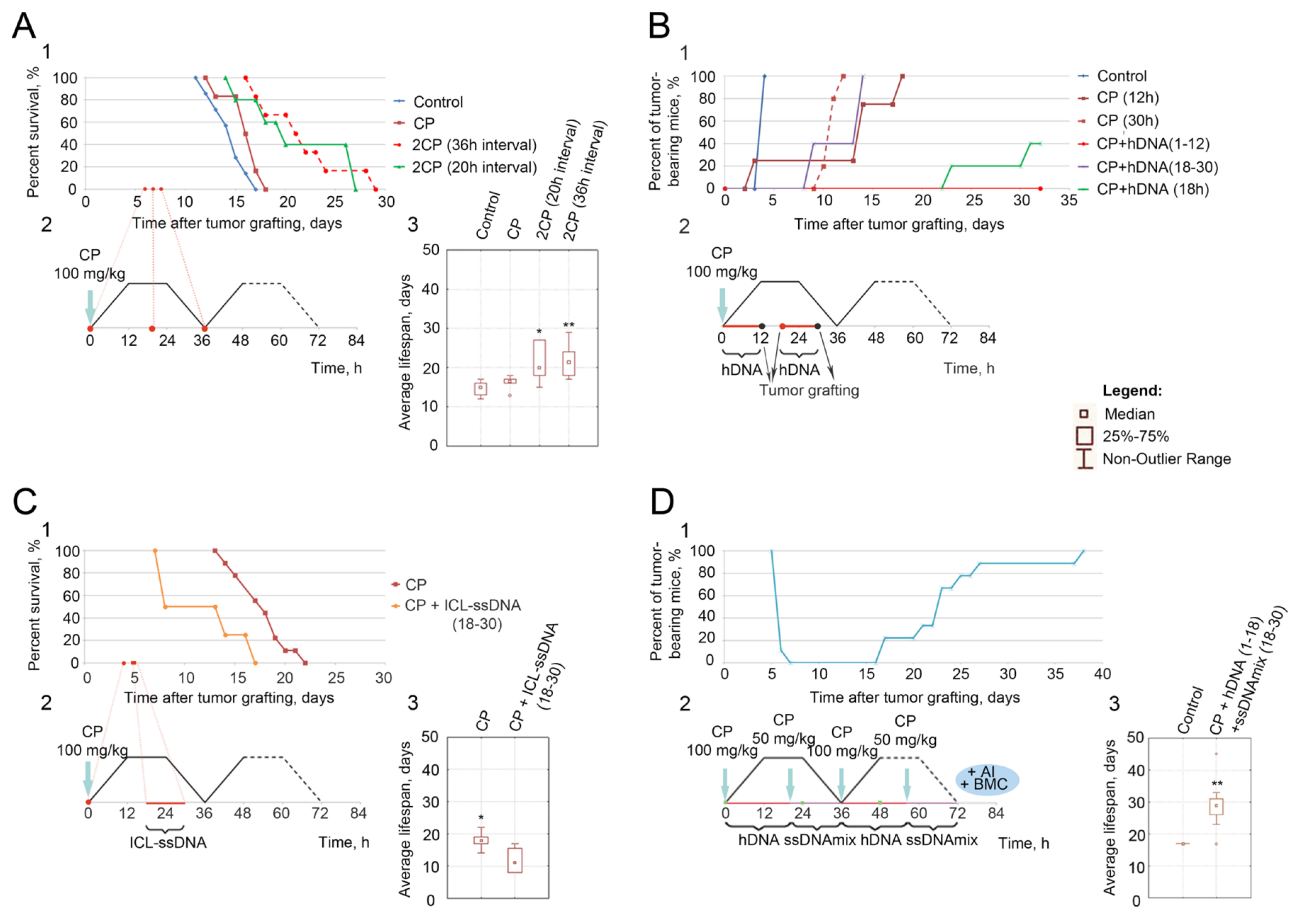
Analysis of therapeutic activity of different injection regimens of CP with or without dsDNA (hDNA, ICL-hDNA, ssDNA, ICL-ssDNA) 1 – survival curve of mice grafted with ascites, 2 – injection schedule, 3 – average lifespan following the treatments, U-test, Wilcoxon-Mann-Whitney, * P < 0.05, ** P < 0.01. **A.** CBA mice bearing 4-day Krebs-2 ascites were treated with CP; **B.** CBA mice bearing 6-7 day Krebs-2 ascites were treated with CP, CP+hDNA 1-12, 18-30 and 18 h post CP (100 mg/kg). I.m. transplantation of tumor cells from treated animals produced solid grafts in recipient mice. The samples labeled as «Control», «CP (12h)», «CP+hDNA (1-12)» and «CP+hDNA (18-30)» had 1.5 mln cells transferred, whereas the rest of the samples contained 0.3 mln cells (compilation of the data published in [2]); **C.** Treatment of CBA mice bearing 4-day Krebs-2 ascites with CP and ICL-ssDNA (18-30 h post CP) compared to the CP-only injection (the data is being prepared for publication); **D.** Analysis of the therapeutic effects of CP combined with hDNA and ssDNAmix on mice bearing 4-day Krebs-2 ascites, followed by BMC transplantation, activation of adaptive immunity [4] and addition of gentamycin (once a day), which helps prevent the development of systemic inflammatory reaction.

Single CP injection given to mice bearing 6-9 day ascites has little if any influence on the development of the grafted ascites transplant [2]. Similarly, a single CP injection into mice with 4-5 day ascites does not affect the survival of animals, as compared to controls (Figure 1A). When CP is given as two injections to mice with 4-5 day ascites at the «zero» timepoint and 20 hours post the first injection (when HR phase is actively inhibited by induction of additional cross-links [new NER]), this results in slower growth of the transplant (Figure 1A). Likewise, two CP injections at the «zero» timepoint and 36 hours later, i.e. when repair process is about to end, also delay the growth of the transplant (Figure 1A). The latter observation can be interpreted as reinforced cell cycle arrest in cells that were at the stage of resolving the first one, and as targeting the now-susceptible subpopulation of cancer cells that were insensitive to the action of CP, as they were in the G2/M phase during the first CP treatment, but have now progressed into G1/S.

### Metronomic injections of dsDNA preparations

Hourly injections of hDNA during the first 12 hours (6 mg total) following the CP injection to mice bearing 6-9 d ascites abrogate the engraftment of ascites. In contrast, the same injections performed during the 18-30 h period post CP treatment produce viable ascites cells whose tumorigenic potential remains essentially unaffected. Hourly injections of ICL-ssDNA during the 18-30 h «window» significantly reduce but not eliminate the engraftment potential of ascites cells [2]. The same holds true for the regimen when a single hDNA injection (6 mg) is given to mice at 18 h post CP timepoint (Figure 1B).

Importantly, hourly injections of ICL-ssDNA (6 mg total) given to mice with 4-5 d ascites during the period 18-30 h post CP injection invariably kill the animals (Figure 1C).

Finally, when mice bearing 4-5 d ascites are first given hDNA (6 mg total) hourly during the first 18 hours post CP injection and then are switched to hourly injections of (ssDNA + ICL-ssDNA) mixture (6 mg total, 18-30 h post CP), ascites in all experimental animals undergo accidental regression. The remission period lasts for up to 5 days, and is followed by a relapse at a secondary site (Figure 1D).

Earlier, we performed comprehensive analysis of cellular and molecular events accompanying the therapy [6, unpublished data] and conclude that the effects observed are based on the selective targeting of Krebs-2 TISCs, which neutralizes the tumorigenic potential of cancer cells [2]. So, we suggested that multiple injections in the metronomic regimen could be substituted by a single injection of a composite dsDNA preparation at the 18 h timepoint post CP (6 mg total) to fully recapitulate the desired therapeutic effect. Notably, this regimen can be repeated several times (accumulation of the «therapeutic dose»), yet it wouldn't be metronomic in its design. Metronomic treatment is non-specific, essentially ignores the biology of the target cells and lacks the exact knowledge to select appropriate timing. In contrast, our regimen targets a well-defined cell subpopulation (TISCs) and is applied during a carefully selected time period.

This novel therapy, which is reduced to a single injection of a composite DNA preparation, may help reduce or avoid the spreading of TISCs into peritoneal space, given that abdominal wall is pierced during multiple injections. Clearly then, this solution should decrease the chances for a secondary tumor to develop at the injection site or elsewhere in the body (the data is being prepared for publication).

Introducing the composite DNA mixture (hDNA, ssDNA and ICL-ssDNA) first at 18 h post CP, followed by two more rounds of CP and DNA (CP given in the beginning of the next repair cycle at 0, 36 and 72 h) should have the following effects:

– Injections of both agents, CP and DNA, will result in enhanced killing of Krebs-2 cancer cells [1, 2, 5, 7].
– Unlike single injections, tri-partite injections of CP at the timepoints matching the beginning of the next repair cycle target all cancer cell subpopulations. These additional rounds of injections progressively affect both committed cancer cells and TISCs that would otherwise remain resistant to CP because they were found at G2/M phase during the preceding CP injection.
– The pilot experiments demonstrated that the ratios of native hDNA/native ssDNA/ICL-ssDNA components in the mixture are optimal for killing ascites cells when used as a single injection.

We expected that:

– ICL-ssDNA would activate NER pathway essentially mimicking the second post CP injection. Re-induction of NER on the background of on-going HR (18 h after first CP injection) appropriately results in the conflict of the two pathways; this kills a fraction of TISCs and reduces the tumorigenic potential of ascites.
– Furthermore, ICL-ssDNA would also interfere with the very onset of HR, and so it will block all the subsequent attempts of the cells to restore the integrity of their DNA via this pathway.
– In turn, at 18 h the hDNA component should come to the spotlight and interfere with the completion of NER phase thereby reducing or completely eliminating the tumorigenic potential of Krebs-2 ascites.
– Importantly, the properties of native hDNA and ssDNA should complement each other in that native ssDNA should competitively prevent the native hDNA from allowing correct completion of HR. Otherwise, hDNA would rescue TISCs from undergoing aberrant mitosis, and so the tumor would survive. Mixing ssDNA and hDNA together is supposed to block the toxicity associated with ICL-ssDNA, whose exact mechanism presently remains unknown.

The concept of using this composite dsDNA-based drug has been developed. Administering the dsDNA preparation exactly during the NER-to-HR transition (18 h post CP) assures that the individual components target the final steps of NER as well as early HR events. In so doing, TISCs are prevented from correctly completing the ICL repair. These cells either die or lose their tumorigenicity, and so does the tumor. Hence, repeating this procedure by exactly following the scheme may result in curing the experimental animals from Krebs-2 ascites cancer.

**Figure 2:**
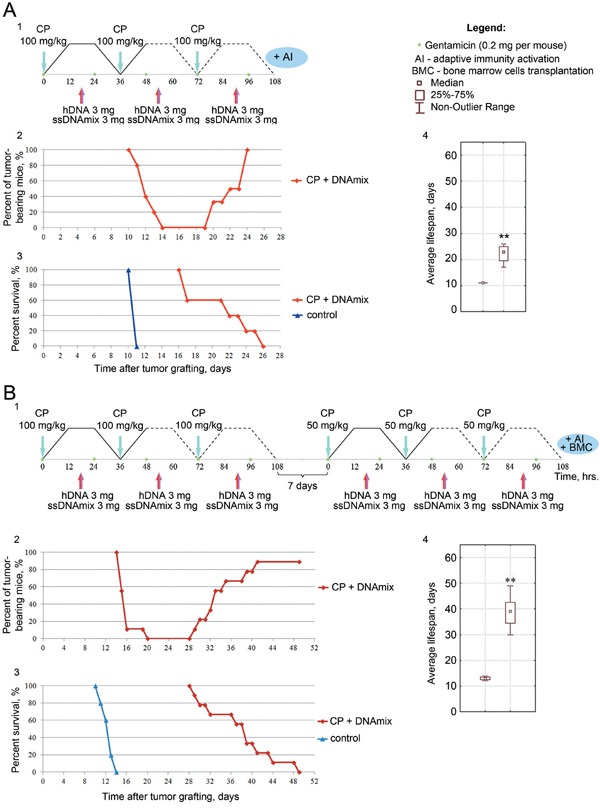
Analysis of a synergistic activity of CP and hDNA+ssDNA+ICL-ssDNA mixture against the ascites form of Krebs-2 tumor in mice 1 – injection scheme; 2 – plot showing ascites resorption, remission and relapse; 3 – survival plot; 4 – analysis of average lifespan in groups of mice, U-test, Wilcoxon-Mann-Whitney, ** P < 0.01. **A.** Synergistic activity of CP and dsDNA, when administered as «3xCP (100 mg/kg) +DNA»; **B.** Synergistic activity of CP and dsDNA, when administered as «3xCP (100 mg/kg) +DNA» followed by a repeat 7 days later with «3xCP (50 mg/kg) +DNA», BMC transplantation, activation of adaptive immunity [4] and gentamicin (daily), - which should prevent the development of systemic inflammatory reaction.

To test this strategy, we treated the mice bearing 4-5 day ascites with composite DNA preparation (a mixture of hDNA + ssDNAmix, 3 + 3 mg/animal, 6 mg total) trice, at 18 h post each of the three CP injections at timepoints «0», and at the beginning of repair cycles (36 and 72 h). This therapy resulted in complete regression of ascites and 5-day remission. However, all mice eventually relapsed and secondary cancer developed (Figure 2A).

We thought that repeating this treatment when animals are in early remission (∼ day 11 following ascites engraftment) may prevent the relapse. We reduced the CP dose during this second round to 50 mg/kg to avoid possible overdosage. All mice nevertheless relapsed, however they did so significantly later, and so the average survival increased (Figure 2B). This result was indicative of the enhanced anti-cancer effect of this therapeutic regimen.

Our studies also pointed to the scenario that some tumorigenic cells invariably survive the treatments and these may likely correspond to TAMRA+ TISCs [2]. So we explored how these cells are affected by the treatments.

### Analysis of TAMRA+ Krebs-2 ascites cells post CP treatment and in the absence of any treatments. Effects of CP+dsDNA treatments on TAMRA+/CD34+ ascites cells

Here we evaluated several parameters of TAMRA+ cells in the native ascites, TAMRA+/CD34+ cells in the ascites treated with a single injection of CP, TAMRA+/CD34+ cells in the ascites treated with a single injection of CP combined with dsDNA, and TAMRA+ cells from ascites of animals that received three CP injections.

**Figure 3:**
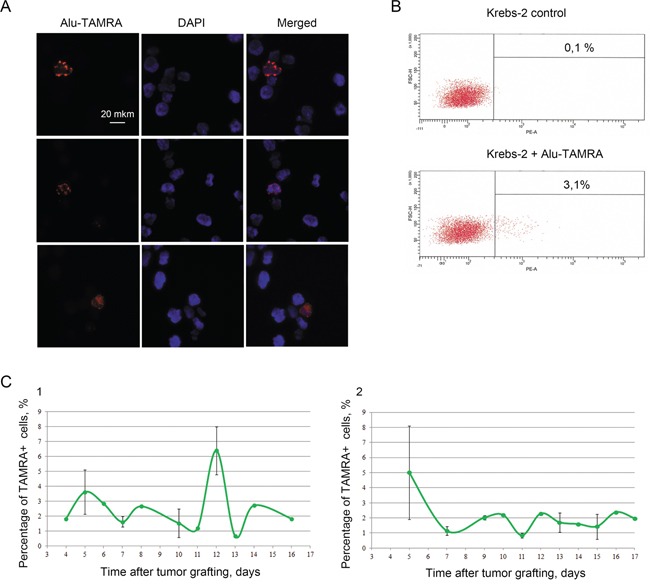
Cytofluorescence A. and flow cytometry B. analyses of *Alu*-TAMRA DNA internalization by TISCs present in Krebs-2 ascites **C.** (1, 2) – dynamics of TAMRA+ cell percentages during ascites growth. Two groups of mice were monitored (data are represented as mean +/− SD, n=3), one of which (1) displayed abortive peak of TAMRA+ cells on day 12.

As ascites grew in animals, we observed that the percentage of TAMRA+ cells remained relatively unchanged (0.5-3.0%, up to the day 15) (Figure 3A, 3B), whereas cell counts increased from 2×10^8 to more than 10^9, and the ascites volume changed from 1 ml to ∼20 ml. Percentage of TAMRA+ cells oscillated throughout the course of ascites growth (Figure 3C).

First and foremost, we wanted to understand what is the efficiency of TISCs elimination by the combined CP+dsDNA treatment, as these cells are known to internalize dsDNA (and so become TAMRA-positive). We could not use a therapeutically meaningful dose of dsDNA to trace the DNA-internalizing cells, as it would outcompete the TAMRA-labeled DNA probe (the data is being prepared for publication). Clearly, use of just TAMRA-labeled DNA would lack the therapeutic effect. To overcome this difficulty, we turned to our earlier studies where we used an additional cell surface marker, CD34. We showed that 40% of cell population capable of internalizing TAMRA-DNA was also CD34-positive and 40-90% of CD34+ cells were TAMRA+, too [2]. So, if the treatments lead to decrease in CD34+ cells, this may indicate that either TAMRA+ TISCs have been eliminated or the surface expression of CD34+ has disappeared. Along with this, we must also take into account the formal possibility that the above treatments may simply target CD34+ TAMRA-cells.

**Figure 4:**
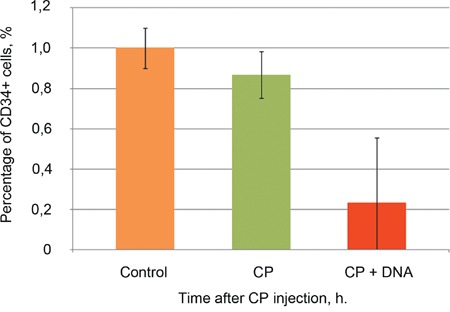
Quantification of CD34+ cell dynamics of Krebs-2 mouse ascites 24 h following CP or CP+dsDNA treatments, FACS analysis «CP» - single CP injection (200 mg/kg); «CP+DNA» - CP injection (200 mg/kg) followed by 12 h DNA injections (hourly, 0.5 mg/injection); «Control» - intact ascites. Data are represented as mean +/−SD, n=3, experiments run in triplicate.

We proceeded with the metronomic scheme of hDNA injections during the first twelve hours post CP (hourly). This choice was dictated by the effect described in Figure 1B, namely by the observation that the tumor graft was rendered completely non-tumorigenic by this treatment. Multiple experiments monitoring the dynamics of CD34+ cells in the treated ascites were performed, and the results of one of these experiments CP+hDNA (1-12) are presented in Figure 4.

We conclude from these experiments that CP treatment goes essentially unnoticed by the CD34+ cell population (at 24 h timepoint), with cell counts staying unchanged. Combining CP with hDNA results in 4-5 fold decrease in CD34+ cell counts (from 1.0 to ∼0.2%) (Figure 4), and this is never accompanied with total elimination of CD34+ cells. This could be interpreted as either overall stronger effects of CP+dsDNA treatment or that dsDNA-internalizing cells (that are also CD34+) are more sensitive to this treatment. Treatment of Krebs-2 ascites under the CP+hDNA (1-12) regimen results in the elimination of TAMRA+ cells, alternatively it may alter their functional state, either way this is manifested as fewer CD34+ cells in the population. Thus, taking into account the results of ascites re-engraftment experiments, as well as the flow cytometry analysis of CP and CP+hDNA effects on TAMRA+ (TAMRA+/CD34+) Krebs-2 TISCs, we come to the following major conclusion: CP is a pre-requisite for terminal eradication of TISCs from Krebs-2 population.

CP is known to result in cell cycle arrest in G1/S. It is also widely accepted that ICLs in G2/M-residing cells remain unrepaired until the next S phase, where they become detected by the cell surveillance machinery and undergo repair (for further references, see [8-10]). It has been reported many times that a single CP injection, even at the highest tolerable dose, has little if any effect on ascites growth, and so some cells important to the tumor growth appear unaffected by such treatment. Yet, CP is also known to result in the massive apoptosis of cancer cells [5]. These two somewhat contradictory facts are nicely reconciled if one assumes that there exists a particular cell subpopulation (TISCs) that is invariably insensitive to CP (regardless of whether the cells are in G1/S or G2/M, as they may be particularly repair-proficient). Adding dsDNA to the CP treatment when these cells are in G/S1 may potently interfere with the repair and so it targets TISCs to destruction. Apparently, not all TISCs are targeted, and the ones found in G2/M evade the toxic activity of both CP and CP+DNA treatments. Clearly then, repeating the treatment when these cells regain sensitivity is key to successful elimination of their entire subpopulation. Such treatment will be accompanied with a second arrest of cells in G1/S, and ICLs will be induced in the cells that were previously resistant to the treatment. Triple treatment in all likelihood will be enough to cover the entire TISCs subpopulation and should be highly detrimental to these cells.

**Figure 5:**
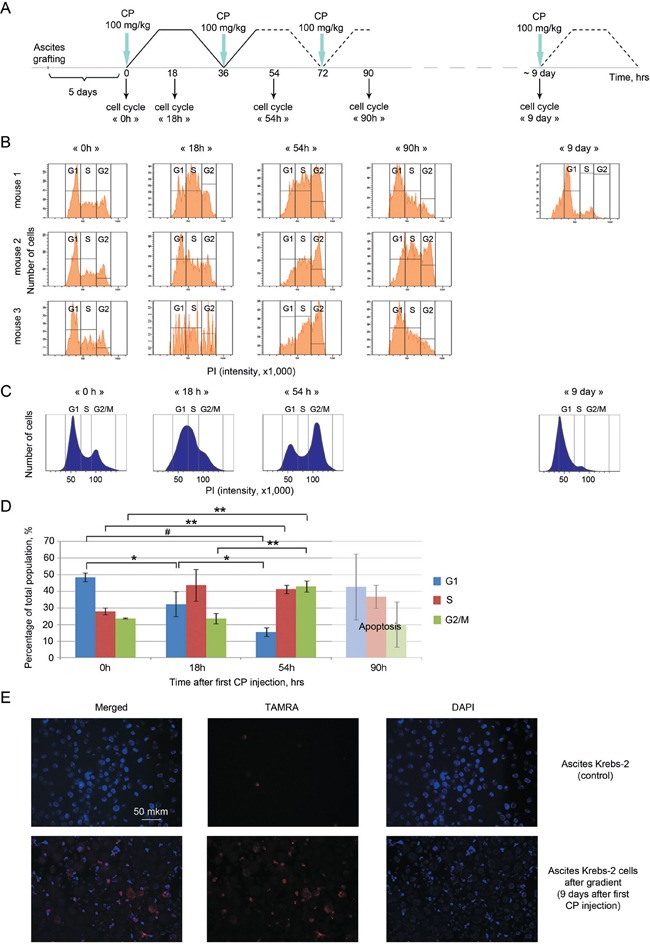
Cell cycle profiling of Krebs-2 ascites from mice that received the indicated therapies **A.** Experiment flowchart. Solid and dashed lines correspondingly denote experimentally verified and tentative phases of NER and HR following CP-induced ICL repair; timepoints 0 h, 36 h, 72 h and 9 d denote CP injections; 0 h, 18 h, 54 h, 90 h and 9 d – ascites cells collection timepoints (followed by FACS profiling); **B.** Cell cycle profiles of ascites cells collected from three individual mice at timepoints 0 h, 18 h, 54 h, 90 h and 9 d (experiment repeated twice); **C.** Schematic drawing of a cell cycle shift towards S/G2/M phase in cancer cells at zero timepoint, 18 h and 54 h after the start of the experiment followed by the synchronous exit of the surviving cells into the G1/S phase on day 9; **D.** Data combined for three animals. Significance of differences was estimated using one-way ANOVA, * P < 0.05, ** P < 0.01, # P < 0.001; **E.** Quantification of TAMRA+ cells in the surviving cancer cells population subjected to concentration on the ficoll-verografin gradient.

Our cytometry analysis of CP-treated cells indicates that consecutive CP injections during specific timepoints result in their arrest in the S phase at 18 h and in shifting of the arrest into late S/G2/M at 54 h timepoint. By the 90 h, the clear-cut DNA-content differences between the cells are no longer visible, and cells tend to be gated out into the apoptotic debris (Figure 5B). By days 7-9, fragmentation of the vast majority of ascites cells is observed. Notably, most of the cells that remain undamaged are in the G1/S phase (Figure 5B, 5C).

The surviving population of cancer cells was concentrated on the ficoll-verografin gradient, and it displayed significant enrichment with TAMRA+ cells: we estimate that there were 12.9% TAMRA+ cells, which is 4-10 times more than what is typically observed for the untreated ascites (Figure 5E).

**Figure 6:**
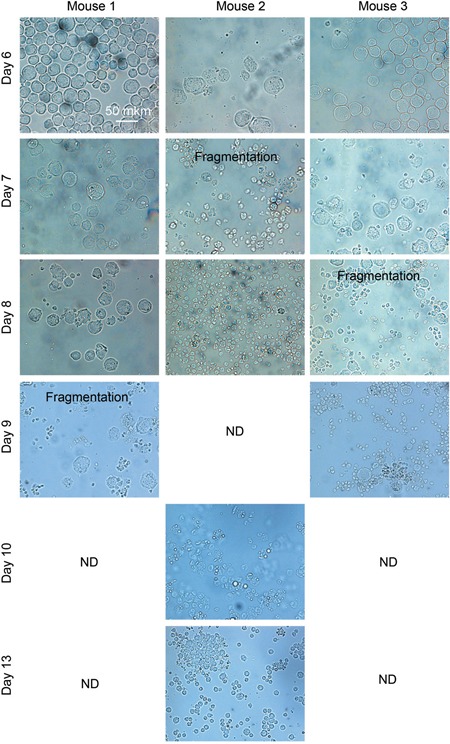
Microscopy analysis of Krebs-2 cells following the treatments indicated in Figure 5A Microscopy analysis of ascites cells collected from three individual animals; fragmentation of cells occurs on days 7-9 after the start of experiment. ND – not detected.

Taken together, these data suggested that a large pool of Krebs-2 ascites cells have undergone apoptotic degradation (Figure 6), whereas the remaining cells (including TAMRA+ TISCs) have become synchronized and have massively progressed into the treatment-sensitive G1/S phase of the cell cycle. This cell degradation state lasts for 2-5 days and is accompanied by higher LDH levels and visual as well as experimentally confirmed resorption of the ascites (the data is being prepared for publication). By day 17, the ascites recovers back to the original level. The percentage of TAMRA+ cells returns to the normal range ∼0.5-3.0%. Thus, much as in the situation of a single CP injection, triple treatment does not eliminate the tumorigenic potential of the ascites and so the tumor eventually relapses (the data is being prepared for publication).

### Regimen for the eradication of Krebs-2 ascites from mice

**Figure 7:**
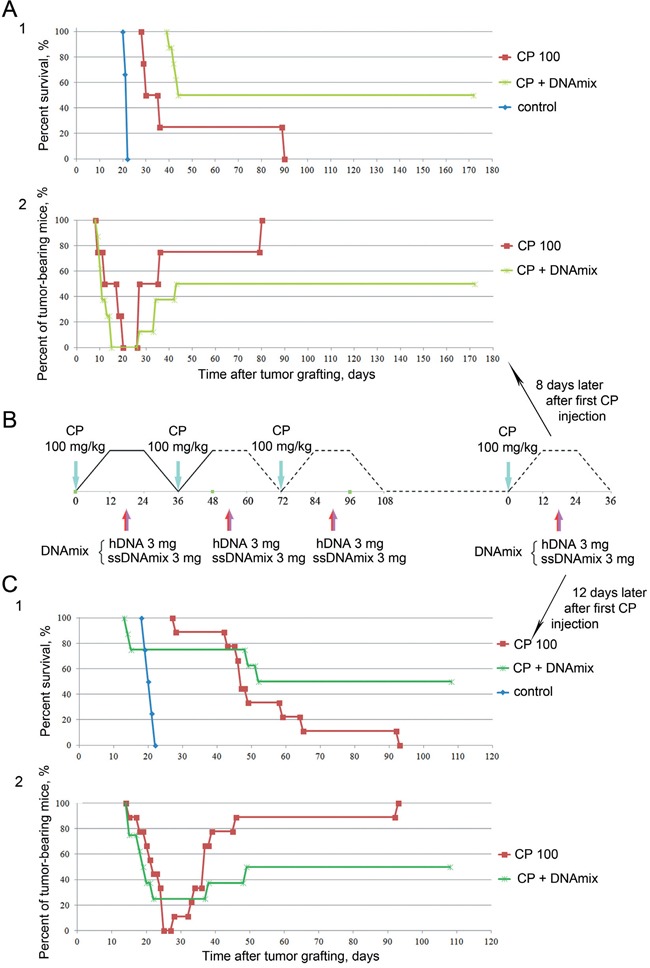
Analysis of a synergistic activity of CP and hDNA+ssDNA+ICL-ssDNA composite mixture towards the well-developed Krebs-2 ascites 1 – animal survival plot, 2 – dynamics of regression, remission and relapse in the treated mice. **A.** Synergistic activity of CP and dsDNA preparation when used as a «3xCP+DNA + final (CP+DNA)» scheme; **B.** Injection schedule for experiments shown in A and C; **C.** Synergistic activity of CP and DNA preparation when used as a «3xCP+DNA + final (CP+DNA)» scheme.

The above studies were highly suggestive of the idea that the timepoint when all the surviving TISCs synchronously enter the G1 phase and so become sensitive to the treatments is critical to the successful therapy. This «final shot» must be done exactly during this period, otherwise the cells will become de-synchronized and will evade the therapeutic attack. To test this hypothesis, we performed two large-scale experiments. The results of these experiments are summarized in Figure 7.

As it turned out, this idea is correct, and 50% of the treated mice can be completely cured. Both CP monotherapy (4 injections) and a combination therapy (CP+dsDNA, 4 injections) displayed pronounced therapeutic effect on the growth of ascites. In the first experiment, the final CP+DNA treatment was performed on day 8 following the first CP injection (i.e. on day 12 after ascites grafting) (Figure 7A, 7B). All mice from CP group developed secondary ascites and eventually all of them died by day 80. Of mice that received CP+DNA, 50% were alive on day 180 (Figure 7A, 7B).

Histopathology analysis of tissues and organs of moribund mice from treated and control groups indicated that the former displayed much weaker peritoneal canceromatosis, no free-floating cancerous cells were present in the abdominal cavity, nor the organs were encased by the cancer cells. In contrast, the control group animals had typical canceromatosis, with tumor cell dissemination throughout the peritoneum. Cancerous nodules of varying sizes were present on the liver, spleen, duodenum, colon, and pancreas (Annex 1).

In mice from the CP-treated group, who developed smaller ascites or lacked ascites altogether, most of the pathological changes were confined to the lung tissue. Three animals from this group were shown to have metastatic growth in the lungs. Additionally, in one of these animals we observed pronounced swelling and necrosis of pancreatic acini due to obstruction of excretory ducts. One animal from the CP+DNA group had the ascites fully resorbed, yet it died in the absence of visible pathologies (Annex 1).

When repeating the experiment, the final round of therapeutic injections was administered on day 12 after the first CP injection (day 16 after ascites engraftment), i.e. four days later than in the first experiment (Figure 7B, 7C). This was done in order to obtain an estimate of how long the fourth CP+DNA treatment may continue to exert ascites-eradicating activity. Much as was described for the first experiment, pronounced therapeutic effect was observed in both experimental groups. Importantly, strict adherence to the therapeutic doses of CP (3×100 mg/kg + 100 mg/kg) and DNA (3×6 mg + 6 mg) was a prerequisite for the effect to occur. Reducing the dosage of either CP or dsDNA, even if injection schedule was maintained, prolongs the time to relapse, but fails to cure the animals (Figure 2B).

In the second experiment, all CP-treated animals succumbed to secondary cancer by day 90. In contrast, 50% of CP+DNA-treated mice remained tumor-free on day 120. Much as in the first experiment, the surviving female mice were mated with males (1×4). In two months, two of the mated females from both experimental arms gave birth to apparently healthy pups – 7 and 6, respectively. One of the females ate her newborn pups on day 3, likely due to the lack of milk. The litter of the second female also died, likely due to the lack of milk, as well. This mouse later produced a second litter, and pups grew and developed normally.

**Figure 8:**
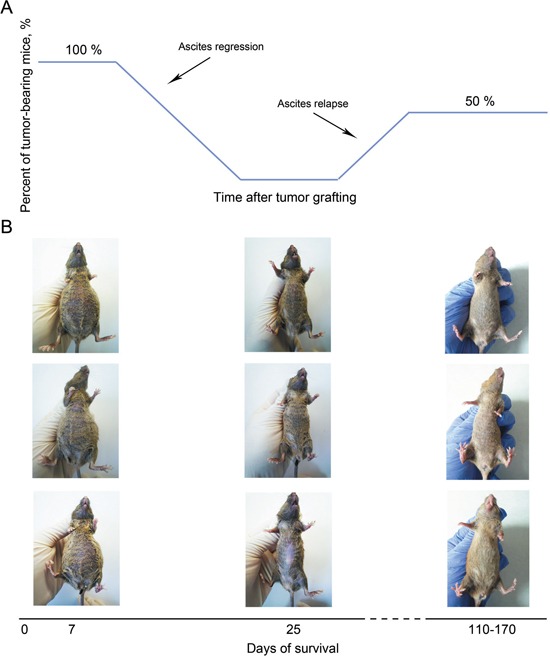
Overview of the therapeutic strategy to treat mice bearing Krebs-2 ascites using CP and dsDNA preparation **A.** Regression of the primary ascites and growth of a secondary ascites in experimental animals subjected to the treatment, up to 170 days post the beginning of therapy; **B.** Mice before the therapy (left panels), during ascites regression period (∼day 25 post the first CP injection, middle panels) and cured mice on day 150 of the experiment (right panels).

Death of experimental animals was caused by two major reasons, one being the developing secondary tumor, the other being uncontrolled multiorgan failure (the data is being prepared for publication). Of 50% of mice that could not be cured, multiorgan failure accounted for about 25-30% of deaths. Experimental pipeline and appearance of the animals throughout the experiments are shown in the Figure 8.

## DISCUSSION

### Features of TAMRA+ TISCs in Krebs-2 tumors

Krebs-2 TAMRA+ cells were previously reported to be highly tumorigenic (i.e. they represent *bona fide* TISCs) [2]. In the present study, we show that the percentage of TAMRA+ cells typically ranges 0.5-3.0%, and it varies slightly in course of ascites growth. In some animals, we observed oscillation in TAMRA+ cells content with a periodicity of ∼4 days, with the largest change from 0.8 to 2.4% and back (Figure 3C2). In a second group of animals, we observed a sharp increase in TAMRA+ cells to 6.4%, which returned to the baseline values soon thereafter (Figure 3C1). In a third group of animals, a pronounced decline in TAMRA+ cells (down to 0.3%) was evident on day 10, followed by return to average values two days later (data not shown). Relative stability of TAMRA+ cells counts may point to the fact that after several rapid cell divisions (the data is being prepared for publication) the progeny of these cells loses the ability to internalize TAMRA-labeled DNA probe. This in turn indicates that for the cancer cells community to maintain the status quo, only 0.5-3.0% of TAMRA+ TISCs are needed, and so there must be a certain ratio between cancer cells and «driver» (TAMRA+) cells to maintain ascites cellular homeostasis.

TISCs are known to divide symmetrically to produce two pluripotent daughter TISCs or asymmetrically producing one TISC and one committed daughter cell. The latter cell has a finite, yet quite significant proliferative potential, and gives rise to many differentiated cancer cells [11-14].

As it follows from quantification of TAMRA+ cell dynamics, these cells may be somewhat heterogeneous in terms of their stemness. Apparently, true TISCs are represented by a rare subpopulation of TAMRA+ cells (below 0.2%, i.e. about 10^6 cells in different animals). The rest of the TAMRA+ cells (∼0.5-3.0%) are likely represented by the first-line committed progeny with high proliferative capacity. After several rounds of cell division, such cells lose the ability to internalize DNA and become differentiated. This ratio (∼0.5-3.0%) is maintained as long as ascites grows. One can hypothesize that TAMRA+ TISCs during the first few days undergo a series of symmetric divisions and so give rise to the required population of «driver» cells. This fixed percentage of TISCs may likely be a universal feature of all malignant tumors, and it may reflect the cell number/tumor volume ratio which allows the regulatory molecules secreted by TISCs to control the entire cell population of the tumor.

### Summary of the developed therapeutic strategy to achieve complete eradication of Krebs-2 ascites

Which molecular and cellular events occur when using the 3+1 protocol of CP+dsDNA injections? Each of the subsequent CP injections fall within the 36 h timepoint, when some of the TISCs that were in G2/M phase and so remained shielded from CP, move to the G1/S thereby becoming CP-sensitive. Each of the subsequent CP treatments leads to an arrest in late S/G2/M of the cells that were trying to repair the damage from the previous treatment. In other words, subsequent CP treatments induce additional cross-links in the cells that have just been repaired, and it prevents such cells from safely progressing through mitosis. Such treatment results in a massive synchronization of both committed cancer cells and residual Krebs-2 TISCs in the late S/G2/M phase (Figure 5B-5D). This synchronization is particularly obvious for the surviving ascites cells on day 9. Most of the cells at this time are in G1/S, with 12.9% of undamaged cells being TAMRA+ (Figure 5E).

When DNA is included in the treatment scheme, this must be done exactly when the cells switch from NER to HR, for the DNA to interfere with both pathways at a time. On days 7-9 post CP, massive destruction of ascites cells is observed: committed cells become apoptotic. The remaining surviving cells (of which 12.9% are TISCs) are uniformly found in G1/S and so they regain sensitivity to the therapy. The final treatment of G1/S-resident cells with CP+DNA kills such cells; therefore no cells with tumorigenic potential are left to proliferate. This scenario is partially supported by the data on the lack of secondary site metastases. Importantly, as much as 50% of the treated animals remain cancer-free long-term, and this is achieved without additional efforts such as immunocorrection and anti-inflammatory/antibacterial therapies. The therapeutic «window of opportunity» for the final CP+DNA treatment is within 8-12 days post the first CP treatment. Pathology analysis indicates that the surviving mice do not display significant organ or tissue damage, which calls for testing this protocol in human trials (Annex 1). Not to be ignored is that only half of the treated animals survive. This may be explained by the following reasons. First, we observed that the phase of massive cancer cell destruction could be shifted +/−2-3 days in the individual animals (Figure 6). So, when following the averaged schedule (days 8 or 12 post CP) ascites in some animals may have already become insensitive or are not yet sensitive to the treatment. Such animals will invariably relapse and die. Multiple organ failure may also be a contributing factor, as it is observed in all the experimental groups, and may require additional therapeutic intervention. We tested the maintenance of fertility in the treated females, and so the therapy does not appear to negatively affect germinal cells. Nonetheless, death of the newborn pups within the first three days, which we attribute to the lack of milk in their mothers, may argue for some physiological problems associated with the therapy. This will require further analysis.

## CONCLUSION

We characterized the therapeutic regimen that allows for 50% of Krebs-2 bearing animals to be completely cured – in spite of the fact that Krebs-2 is generally considered a highly malignant and essentially incurable form of cancer. Our analysis narrowed down the timepoints when the therapy is effective, and the entire strategy is based on the biology of Krebs-2 TISCs. First, these cells are naturally capable of internalizing fragmented DNA. Second, such DNA fragments may potently interfere with the ongoing repair of ICLs induced by an earlier CP treatment such that the TISC either dies or is no longer tumorigenic.

## MATERIALS AND METHODS

### Lab animals

We used 2-8-month old CBA/Lac and C57Bl mice strains (males and females, 20-25 g) bred at the Institute of Cytology and Genetics, SB RAS. Animals were grown in groups of 5-10 mice per cage with free access to food and water. All experiments were performed in accordance with the protocols approved by the Animal Care and Use Committee of the Institute of Cytology and Genetics.

### Tumor model

Ascites form of a murine carcinoma Krebs-2 (derived from the solid form) was used as a model [3]. This cancer cell line was obtained from the cell depository of the Institute of Cytology and Genetics (Novosibirsk, Russia) and is maintained in mice as a transplanted tumor. To obtain ascites-bearing mice, Krebs-2 cells were adjusted to 10^7 cells/ml and inoculated intraperitonially (i.p.) in 200 mkl of normal saline (2×10^6 cells). Ascites growth was monitored by weighting the animals. Ascites remission was scored visually, whenever the abdomen of mice appeared indistinguishable from that of an intact mouse, which was accompanied by the appearance of characteristic stretch marks due to dramatic decrease in the volume of fluid in the abdominal cavity.

To obtain solid tumors, Krebs-2 cells were engrafted intramuscularly (i.m.) into the right hind leg in 100 mkl of RPMI-1640 or PBS. In course of experiments, as soon as tumors became palpable (about 7 days following the inoculation), they were measured every 1-2 days with calipers, and tumor volume was calculated as follows: volume = length × width × height.

### Injection of CP and exogenous DNA preparations

I.p. injections of a cytostatic drug CP (Veropharm, Russia) were given to mice at a dose of 50, 100 or 200 mg/kg body weight. Next, depending on the specific experimental design, the preparations of fragmented human or salmon sperm DNA (hereafter, «hDNA» or «ssDNA») or nitrogen mustard-treated DNA («ICL-hDNA» or «ICL-ssDNA») were injected i.p. into ascites tumor at 0.5 mg/injection (hourly schedule) or at 6 mg/injection («single» injection regimen). The following injection schedules were used: hourly 1-12 h or 18-30 h after CP injection (i.e. a total of 6 mg hDNA prep/mouse) or a single injection of 6 mg hDNA + ssDNA/ICL-ssDNA mix (5/2 or 5/3). As a control, equal volume of normal saline was used.

### Adaptive immunity activation

In order to achieve adaptive immunity activation, hDNA preparation (500 mkg/animal) was injected to mice 1, 2 and 3 days following the final CP injection [4].

### Bone marrow cell transfer

Healthy syngeneic mice were used as donors to obtain bone marrow cells. Bone marrow cells were collected from mouse tibia by perfusion with PBS, washed with RPMI-1640 medium, quantified using hemocytometer and injected into the tail vein of experimental animals at a dosage of 2×10^5 cells/mouse.

### DNA preparation

Human DNA preparations were isolated from placentas of healthy women using a phenol-free method. DNA was fragmented in an ultrasonic disintegrator at a frequency of 22 kHz to obtain a mixture of DNA fragments ranging 200 to 6000 bp in size. DNA preparations were dissolved in saline and stored at −20°C. This is a pharmacopeial drug (product license JICP-004429/08) and is manufactured under the trademark of Panagen, Ltd.

Salmon sperm DNA preparation from salmon milt was obtained essentially the same way.

### Preparation of nitrogen-mustard cross-linked DNA preparation

ICL-DNA preparation procedure was described in [5].

### Histopathology analysis of mouse tissues and organs

Pieces of tissues, organs, tumor foci and peritoneal wall from mice engrafted with ascites were fixed in 4% formaldehyde, dehydrated in a graded series of alcohols, cleared in xylol and embedded in paraffin. 5 mkm thick paraffin sections were stained with hematoxylin and eosin. AxioImager ZI microscope (Zeiss) was used for imaging.

### Internalization of human TAMRA-labeled *Alu*-DNA by Krebs-2 ascites cells

The assay to quantitatively analyze TAMRA-internalizing cells is described in [2].

### Analysis of re-engraftment potential of in vivo treated Krebs-2 ascites

Mice with well-developed Krebs-2 ascites (day 4-7) received injections of CP (100-200 mg/kg) and DNA preparations (6 mg total) according to the treatment schemes. Some time after the treatments, ascites cells were collected from the animals, spun down at 400g for 5 minutes (4°C) and counted using hemocytometer. Suspension of cells in 100 mkl of RPMI-1640 or PBS was injected i.m. into the right hind limb of intact mice. Transfer of varying numbers of cells was tested (0.3-1.5×10^6 cells/mouse). Control animals received matching numbers of untreated Krebs-2 cells.

### Isolation of Krebs-2 ascites cells using ficoll-verografin density gradient centrifugation

Following the therapeutic manipulations, 0.1 ml of ascites was collected from mice, resuspended in 3 ml PBS, loaded on top of 3 ml ficoll-verografin (1.077 g/cm3) mix, and centrifuged for 30 minutes at 1500 rpm. Top interphase was collected by introducing the tip throughout the upper liquid phase, and washed once with PBS.

### Cell cycle analysis of Krebs-2 ascites cells subjected to triple CP injections at 36 h intervals

Krebs-2 ascites were engrafted to mice (2×10^6 cells/animal). The first CP injection (100 mg/kg) was administered on day 5, which was followed by two more CP injections, 36 and 72 h later. Ascites cells were collected at these timepoints to monitor the cell cycle distribution of ascites cells. Small volume (about 50 mkl) of ascetic fluid was collected using a syringe and washed once with PBS. Cells were counted and 6×10^5 cells were fixed in 50% methanol (300 mkl methanol is added dropwise to the cell suspension). When all the samples were collected and fixed, the cells were centrifuged, washed once with PBS, resuspended in 200 mkl PBS and treated for 30 minutes with RNase (200 mkg/ml, 37°C). Next, the cells were re-spun and resuspended in 400 mkl of PBS. Propidium iodide (#P4170, Sigma) was added to the final concentration of 10 mkg/ml. The cells were then analyzed on flow cytometer BD FACS Aria.

### Cell fragmentation analysis

Ascites cells from CP-treated mice were collected at the indicated time points. 1.5×10^5 cells were cytospun on slides and mounted under coverslips in glycerol. Images were taken in transmitted light using Axioscope 2 plus microscope and AxioVision software.

### Quantification of CD34+ cells in the ascites form of Krebs-2 tumor using FACS

Quantification of CD34^+^ cells present in Krebs-2 ascites is described in [2].

### Statistics analysis

Comparisons of average lifespans of animals from different experimental groups were performed using non-parametric statistic analysis (Wilcoxon-Mann-Whitney t-test) embedded in the Statistica package. One way ANOVA was used for the analysis of differences in cell cycle distributions (Figure 5D).

## SUPPLEMENTARY DATA FIGURES


