# Supplementary material for: A strategy to eradicate well-developed Krebs-2 ascites in mice
Source: Oncotarget. 2016 Feb 10;7(10):11580–94. doi: 10.18632/oncotarget.7311 (PMC4905495; doi:10.18632/oncotarget.7311)
Supplement: Supplementary file 1 [file oncotarget-07-11580-s001.pdf]

SUPPLEMENTARY DATA

Histopathology analysis of organs and tissues from ascites-engrafted mice subjected to treatments with CP or CP+dsDNA vs control mice

The present Annex features the results of histopathology analysis of organs and tissues isolated from agonizing mice and control animals. In total, the analysis was performed for two control group animals (No 1 and No 2), three CP group animals, two of which succumbed to the secondary ascites (No 1 and No 2) and the third died because of multiple organ failure (No 3), and one animal from the CP+dsDNA group that did not have obvious ascites but apparently died due to multiple organ failure.

Control animals

Lung tissue

Lung tissue appeared essentially normal, yet it displayed somewhat reduced airness, thickened (swollen) interalveolar walls and small clusters of lymphocytes around the vessels (rarely) (Figure 1A). In the second control animal, these features are much more pronounced, and lymphocytes infiltrate the interalveolar walls over a greater area (Figure 1B).

Lymph nodes

In one animal, large tumorous nest was found sitting on the surface of the lymph node, which was separated by a thin sheath of connective tissue (Figure 2A). In the second animal, cancer cells were also found on the lymph

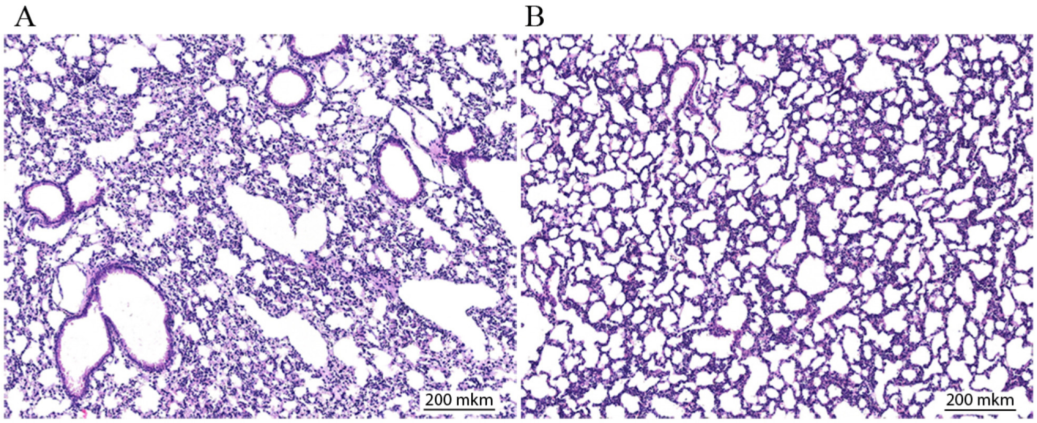

Figure 1: Lung tissue of two control group mice, No 1 (A) and No 2 (B). A. Swelling of interalveolar walls, perivascular clusters of lymphocytes; **B.** Interalveolar wall infiltration by lymphocytes.

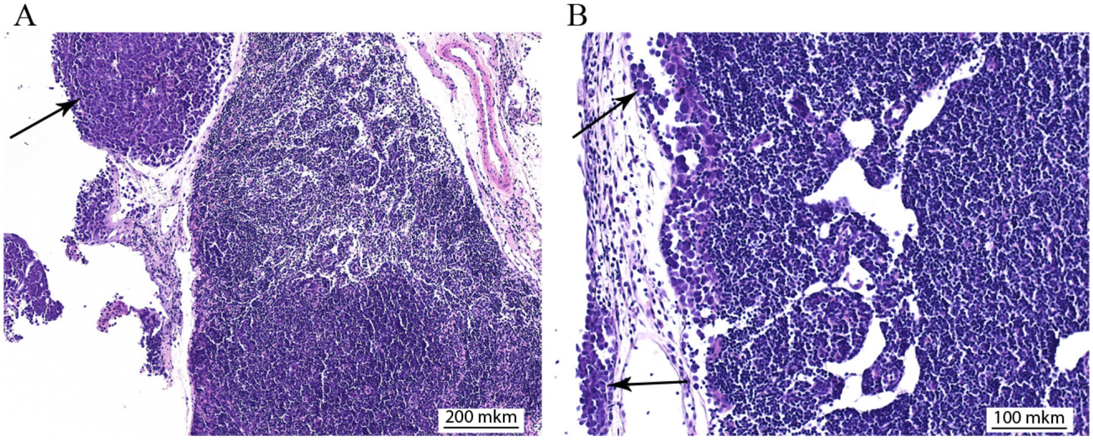

Figure 2: Lymph nodes from the animal No 2 (A) and animal No 1 (B) (control group). A. Large aggregation of cancer cells on the surface of the lymph node (arrow); **B.** Cancer cells on the surface (lower arrow) and in the lumen of marginal sinus lumen (upper arrow) of the lymph node.

node serosa and in the sinus lumen (Figure 2B). Several cancer cells were scattered across the paracortical zone.

Small intestine

Large cancer cell node was found on the intestinal serosa in one of the animals (Figure 3A). The rest of the intestinal cell wall layers, submucous layer, as well as mucosa were normal (Figure 3B).

In the second animal, tumor cells have almost entirely covered the small intestines outer wall (Figure 3C). The cancer nodule displayed small necrotic foci, with cancer cells spreading into the muscular wall (Figure 3D). Intestinal mucosa remained essentially intact.

Large intestine

The situation is very similar to that described for the small intestine. The degree of invasion in the second animal is much higher. Even though the integrity of the mesenteric wall is not compromised, cancer cells are found under the external longitudinal layer (Figure 4).

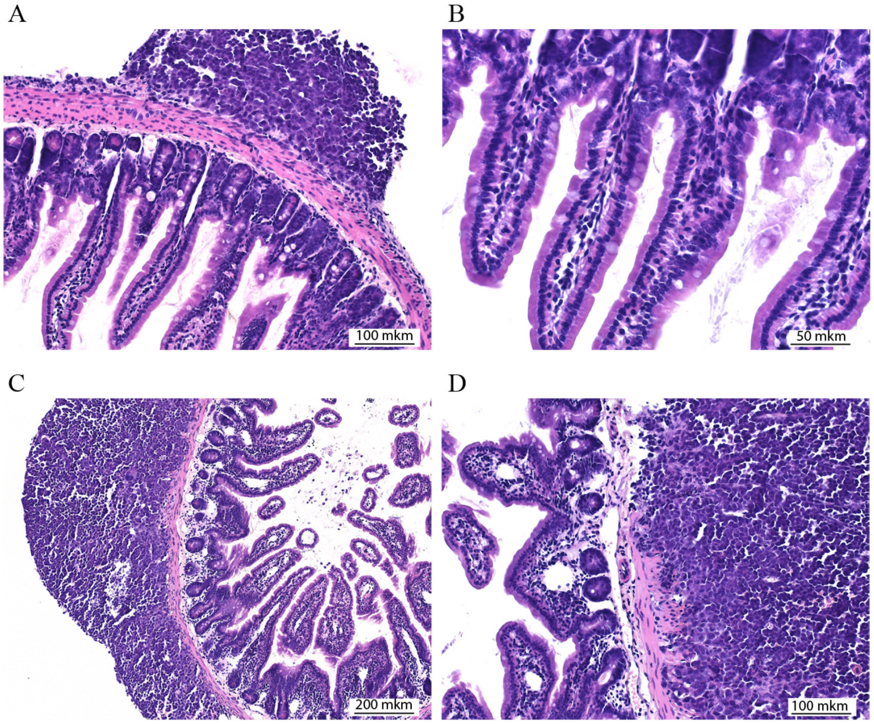

Figure 3: Small intestine of the control group mice: A and B – animal No 1, C and D – animal No 2. A. Cancerous nodule attached to the surface of the small intestine; **B.** Epithelial sheath of the intestine is intact; **C.** Tumor covers the surface of the intestines; **D.** Small intestine. Left– intestinal lumen, right – cancer nodule, center – muscular wall of the small intestine (muscular wall is partially degraded by the growing cancer cells – upper part of the image).

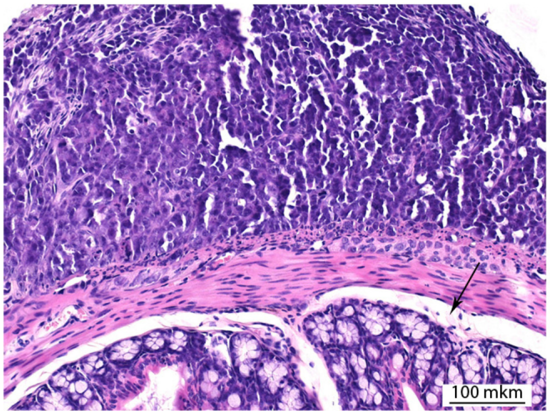

Figure 4: Cancer cells found between the large intestine myenteron layers (arrow). Control animal No 2.

Pancreas

In both animals, we observed that large cancer nodules were present on the surface of the pancreatic capsule. In the second animals, cells of the large cancer nodule were also found to invade the subcapsular space (Figure 5).

Kidneys

No pathologies.

Adrenal glands

No pathologies.

Liver

The first animal in the control group had cancer cells located on the surface of the visceral peritoneum covering the liver (Figure 6A). It invaded under the capsule at several sites (Figure 6B) but never spread beyond the marginal layer. In the second animal, we observed a large cancer nodule with a central necrotic zone (Figure 6C).

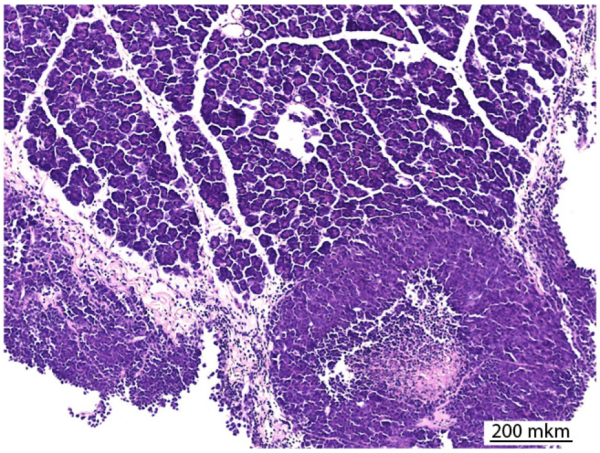

Figure 5: Large cancerous nodule with a prominent necrotic zone is found on the surface of the pancreas. Data for the animal No 1 are shown.

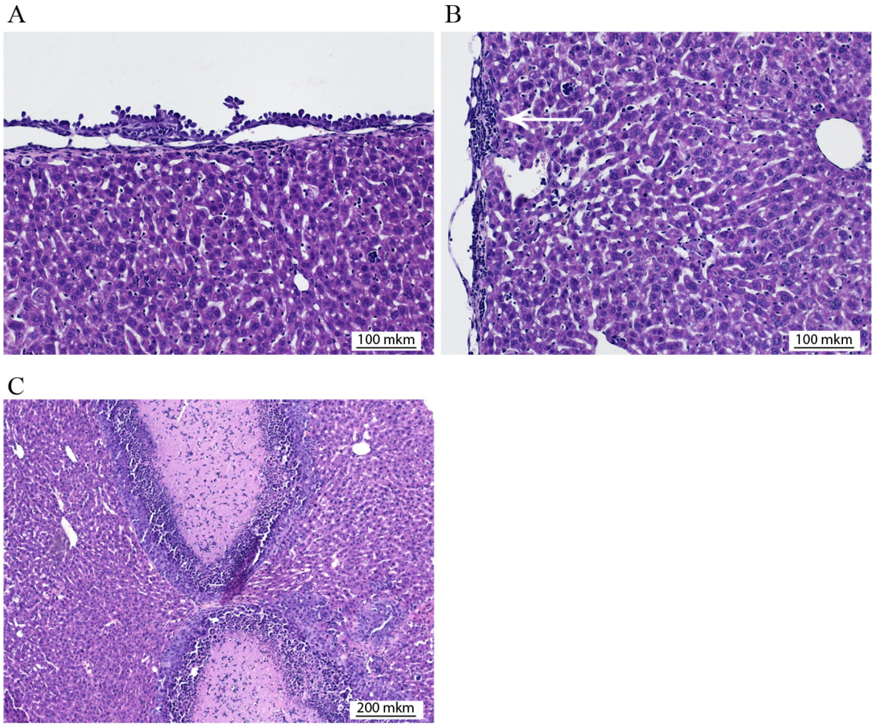

Figure 6: Liver samples from control group animals No 1 (A and B) and No 2 (C). A. Cancer cells attached to the liver capsule; **B.** Cancer cells invade the subcapsular space (arrow); **C.** Cancer nodule with a central necrotic zone found in the liver.

Primary tumor localization could not be unambiguously established, but it definitely was not growing from the surface, i.e. it is likely a metastasis.

Spleen

In both control animals, cancer nodules with or without necrotic zone were found on the splenic capsule (Figure 7).

Cancer nodules

Cancer nodules composed of the aggregated cancer cells were found. Most of them were found attached to the parietal peritoneum lining the abdominal wall. Cancer cells invaded the peritoneum and spread between the muscle fibers of abdominal wall.

CP-treated group

Lungs

In both animals from this group, besides moderately swollen interalveolar walls, we observed clusters of cancer cells, some of which were destroyed (at least four such clusters are visible in the Figure 8A). At a higher magnification (Figure 8B), one can see that these cancer cells are covered with endothelial cells and apparently myocytes. In other words, these structures likely correspond to small lymphatic vessels or to postcapillary venules.

In the animal No 3, lung parenchyma appears consolidated due to interstitial swelling and atelectasis; profuse lymphocytic infiltration and large necrotic zones are visible (Figure 8C). Single cancer cells are frequent in the alveolar openings (Figure 8D).

Lymph nodes

Intact.

Small intestine

Scattered solitary cancer cells were present on the surface of the small intestine covered with peritoneum.

Large intestine

Normal.

Pancreas

In one animal, pancreatic tissue was heavily swallen and acini appeared necrotic. We attribute this to the obstruction in the pancreatic duct (Figure 9).

Kidneys

Normal.

Adrenal glands

In one animal from the CP group, the retroperitoneal cover of adrenal gland displayed solitary cancer cells (Figure 10), which may indicate that the tumor has disseminated through the parietal peritoneum close to the kidney.

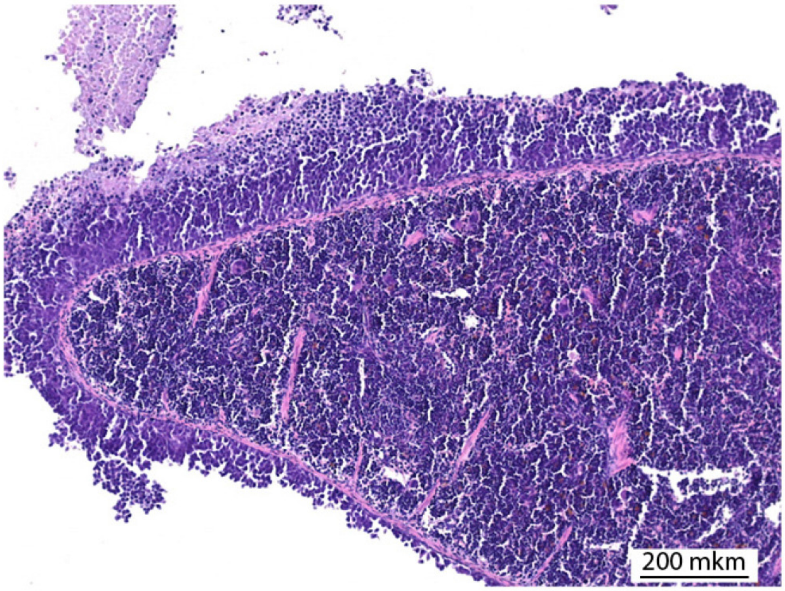

Figure 7: Cancer cells covering the outer surface of the splenic capsule. Animal No 2.

Liver

Normal.

Spleen

Splenic follicles appeared somewhat degenerated.

Cancer nodules

Cancer nodules composed of the aggregated cancer cells (frequently with large regions of necrosis) were found (Figure 11). Most of the nodules were attached to the parietal peritoneum. Cancer cells invaded the peritoneum and spread throughout the muscular layer of the abdominal wall.

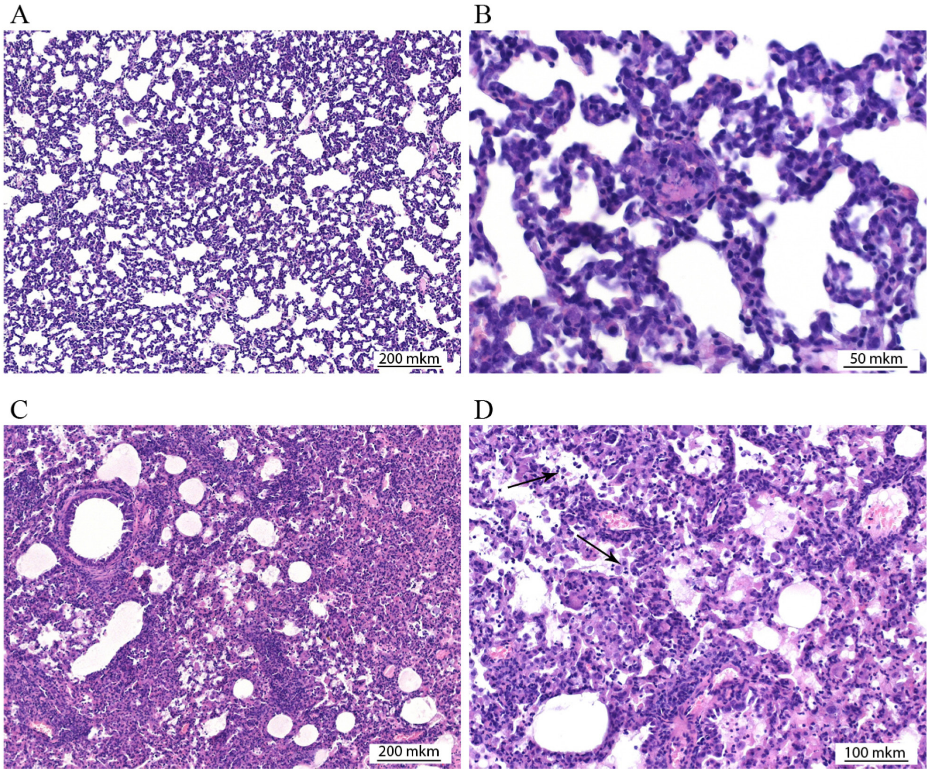

Figure 8: Lung structure in animals from the CP group. A. Swelling of interalveolar walls, clusters of cancer cells present (animal No 2); **B.** Cancer cell cluster surrounded by endothelial cells (animal No 2); **C.** Interstitial swelling and atelectasis; necrotic region is visible on the right hand part of the image (animal No 3); **D.** Cancer cells detectable in the alveoles (arrows) (animal No 3).

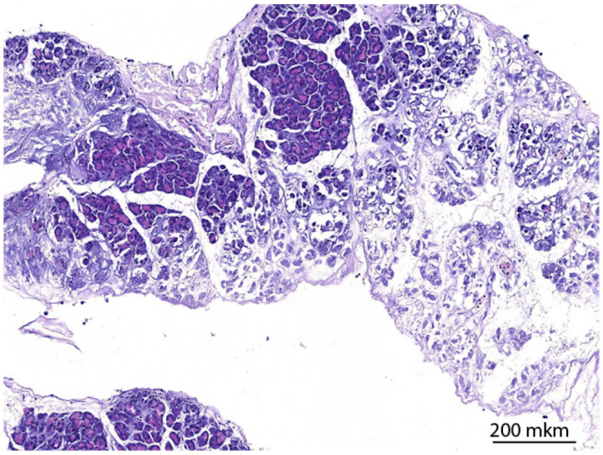

Figure 9: Stromal swelling and acinar cell necrosis in the pancreas (animal No 1 from the CP group).

CP+dsDNA group

Lung

The only CP+dsDNA group animal studied had an intact lung tissue.

Lymph nodes

Intact.

Small intestine

No pathology.

Large intestine

No pathology.

Spleen

No pathology.

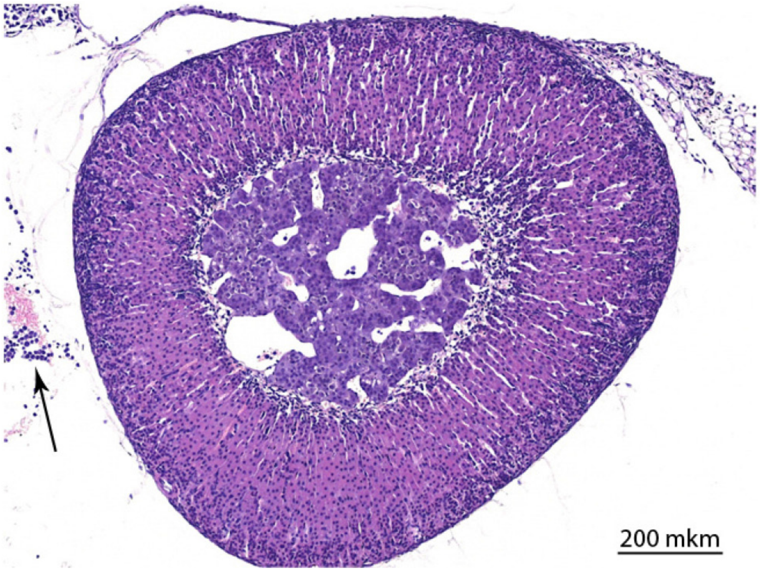

Figure 10: Cancer cells (arrow) present in the retroperitoneal fat surrounding the adrenal gland (animal No 2 from the CP group).

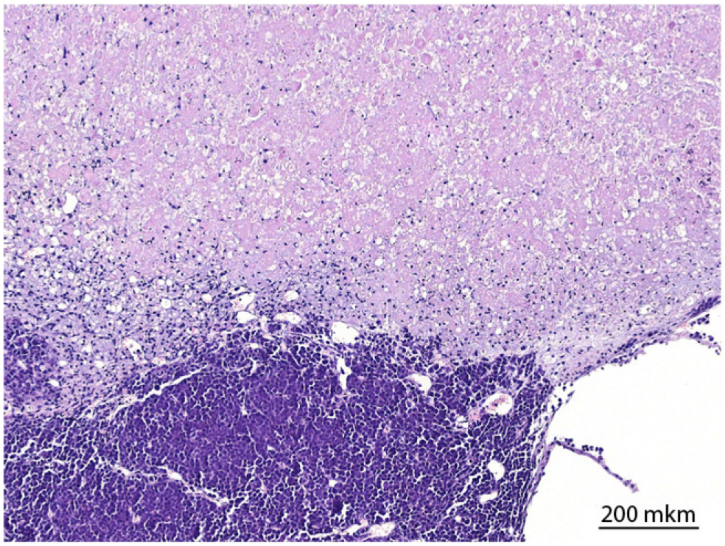

Figure 11: Large necrotic zone (upper part of the image) in the cancer nodule found in the animal No 2 from the CP group.

Kidneys

No pathology.

Adrenal glands

No pathology.

Liver

No pathology.

Spleen

Splenic follicles appeared somewhat degenerated.

Cancer nodules

Absent.

No pathologies were found in the heart and stomach of the animals from either of the groups studied.

CONCLUSION

Animals from the control group displayed the pathologies consistent with canceromatosis, i.e. dissemination of tumor cells throughout the abdominal cavity and attachment to the peritoneum. Cancer nodules typically located to the sites where peritoneum forms recesses, i.e. where visceral pleura contacts parietal pleura. Cancer nodules of varying sizes were present on the surface of liver, spleen, large and small intestine, and pancreas. In one animal a large metastasis in the liver was found – we speculate that it was formed by cancer cells that travelled through the hepatic portal system. We also note cancer cells invasion across the pancreatic capsule and infiltration of a lymph node (in the same animal).

In the CP group, most of the pathologies were restricted to the lungs, which were heavily metastasized. We think that cancer cells ended up in the lungs by travelling through the lymphatic system (as a consequence of invading the lymph node basin and breaking the integrity of peritoneal drain system). In one of the animals from this group, we observed swelling and acinar necrosis of the pancreas, due to obstruction in the pancreatic duct.

The animal from the CP+dsDNA group displayed very few pathology issues, except for a small sclerotic focus under the kidney capsule, which we believe was unrelated to the therapy or disease.

Overall, peritoneal canceromatosis appears much less pronounced in the experimental (treated) groups than in the untreated controls. No cancer cells are found in the abdominal cavity, nor large cancer nodules are present.
